# Sexual Dimorphism in White Matter Developmental Trajectories Using Tract-Based Spatial Statistics

**DOI:** 10.1089/brain.2015.0340

**Published:** 2016-02-01

**Authors:** Kiran K. Seunarine, Jonathan D. Clayden, Sebastian Jentschke, Monica Muñoz, Janine M. Cooper, Martin J. Chadwick, Tina Banks, Faraneh Vargha-Khadem, Christopher A. Clark

**Affiliations:** ^1^ UCL Institute of Child Health, London, United Kingdom.; ^2^ Cluster “Languages of Emotion”, Freie Universität Berlin, Berlin, Germany.; ^3^ School of Medicine, University of Castilla–La Mancha, Albacete, Spain.; ^4^ Child Neuropsychology, Murdoch Childrens Research Institute, Melbourne, Australia.; ^5^ Division of Psychology and Language Sciences, Research Department of Cognitive, Perceptual, and Brain Sciences, Institute of Behavioral Neuroscience, University College London, London, United Kingdom.; ^6^ Great Ormond Street Hospital for Children, London, United Kingdom.

**Keywords:** brain development, diffusion tensor imaging, gender differences, puberty, tract-based spatial statistics, white matter

## Abstract

Increasing evidence is emerging for sexual dimorphism in the trajectory of white matter development in children assessed using volumetric magnetic resonance imaging (MRI) and more recently diffusion MRI. Recent studies using diffusion MRI have examined cohorts with a wide age range (typically between 5 and 30 years) showing focal regions of differential diffusivity and fractional anisotropy (FA) and have implicated puberty as a possible contributory factor. To further investigate possible dimorphic trajectories in a young cohort, presumably closer to the expected onset of puberty, we used tract-based spatial statistics to investigate diffusion metrics. The cohort consisted of 23 males and 30 females between the ages of 8 and 16 years. Differences in diffusion metrics were corrected for age, total brain volume, and full scale IQ. In contrast to previous studies showing focal differences between males and females, *widespread* sexually dimorphic trajectories in structural white matter development were observed. These differences were characterized by more advanced development in females compared to males indicated by lower mean diffusivity, radial and axial diffusivity, and higher FA in females. This difference appeared to be larger at lower ages (8–9 years) with diffusion measures from males and females tending to converge between 10 and 14 years of age. Males showed a steeper slope for age-diffusion metric correlations compared to females, who either did not correlate with age or correlated in fewer regions. Further studies are now warranted to determine the role of hormones on the observed differences, particularly in 8–9-year-old children.

## Introduction

There are widespread structural changes in the brain as it matures throughout childhood, into adolescence, and early adulthood. Over this time frame, there is considerable white matter organization as well as myelination of white matter axons (Yakovlev and Lecours, 1967) and synaptic pruning of gray matter (Huttenlocher and Dabholkar, 1997). Typical findings from brain development studies (Caviness et al., 1996; Giedd et al., 1999; Reiss et al., 1996; Sowell et al., 2003) show an increase in white matter and decrease in gray matter volume.

Although the most significant changes occur up to around the age of 5 years, the brain continues to undergo structural maturation throughout childhood and into adulthood. There is also evidence that gender affects the developmental trajectory. For example, several studies (Giedd et al., 1999; Reiss et al., 1996) have reported gender differences in the proportion of gray matter and white matter of the brain.

Diffusion magnetic resonance imaging (dMRI) is a technique that provides unique insight into the microstructure of brain tissue. Diffusion tensor imaging (DTI) (Basser, 1994) is the standard technique for reconstructing dMRI data and provides several useful metrics. Two of the most commonly used metrics are fractional anisotropy (FA) and mean diffusivity (MD) (Basser and Pierpaoli, 1996). MD describes the amount of diffusion in a voxel regardless of orientation; FA describes the degree of directionality of diffusion. Both measures are widely used as biomarkers of white matter integrity and have been used extensively to investigate the structural development of white matter pathways. In addition, the axial (λ_axial_) and radial (λ_radial_) diffusivities can provide insight into the nature of the diffusion profile (the 3D distribution of diffusion in a voxel, which in this case is represented by the diffusion tensor). Specifically, they can show whether the diffusivity in the direction of the principal eigenvector or the diffusivity perpendicular to it may be driving changes in FA and MD.

Several methodologies have been applied to the investigation of brain development using dMRI, including region of interest analysis (Bonekamp et al., 2007; Hermoye et al., 2006; Kumar et al., 2012; Mabbott et al., 2006; Snook et al., 2005), tractography (Asato et al., 2010; Eluvathingal et al., 2007; Fjell et al., 2008; Lebel et al., 2010; Lim et al., 2015; Tamnes et al., 2010a), and voxel-based morphometry (VBM) (Ashtari et al., 2007; Barnea-Goraly et al., 2005; Giorgio et al., 2008, 2010; Qiu et al., 2008; Schmithorst et al., 2008). In 8–12-year-old children, Snook et al. (2005) detected increases in FA with age in the genu and splenium of the corpus callosum, corona radiata, putamen, and head of the caudate nucleus.

Eluvathingal et al. (2007) used tractography to find age/sex differences in subjects in the age range between 6 and 17 years. They found three patterns of correlation with age: increases in FA and decreases in diffusivity, decrease in diffusivity with no observed change in FA, and no significant changes in either of the measures. In addition, they found that females had a lower radial diffusivity than males in the inferior longitudinal fasciculus bilaterally. In contrast, Schmithorst et al. (2008) used a VBM approach to show significant age–sex interactions as well as local differences in white matter microstructure between genders for children/adolescents. In particular, they found FA to be greater in girls than boys in the splenium of the corpus callosum. They also found FA to be greater in boys than girls in frontal white matter, right arcuate fasciculus, and left parietal and occipitoparietal white matter.

Other studies have shown widespread structural changes across the brain with increasing age. For example, Barnea-Goraly et al. (2005), also using a VBM approach, showed positive correlations between FA and age in the corpus callosum, prefrontal regions, basal ganglia, internal capsule, and thalamic pathways in children and adolescents aged 6–19 years. More recently, Lim et al. (2015) used structural graph connectivity to investigate normal brain development in a cohort of 121 participants aged 4–40 years. They not only observed that the core features of the connectivity networks, such as small-worldness and modular organization, remained similar throughout development but also found that streamline count reduced with age for specific types of fiber tract. Furthermore, they observed that reductions in streamline count occurred earlier in females than males.

Tract-based spatial statistics (TBSS) (Smith et al., 2006) is a voxel-based technique specifically developed for DTI data. Unlike other voxel-based methods, such as VBM, this technique performs statistical analysis on an FA skeleton that comprised a subset of the image voxels, which avoids the need to smooth the data and also reduces problems caused by partial volume effects. Giorgio et al. (2008) performed a TBSS study on children/adolescents and young adults and showed significant positive correlations between FA and age in the body of the corpus callosum and the corona radiata in the childhood/adolescent group. More recently, Giorgio et al. (2010) performed a longitudinal TBSS analysis on DTI data in children and adolescents in the age range 13.5–18.8 years (mean follow-up 2.5 years). In contrast to their previous study, they showed significant increases in FA between time points across the FA skeleton. Tamnes et al. (2010b) used TBSS in a study of 8–30-year olds and showed that both verbal and performance abilities were positively related to FA and negatively related to MD and λ_radial_ predominantly in the left hemisphere.

The aim of the present study was to further elucidate possible differences in the trajectory of white matter development in males and females. Asato et al. (2010) have shown using TBSS that maturation of white matter in specific brain regions is earlier in females compared to males in a cohort spanning childhood through to adulthood from 8 to 28 years. The observation of an effect of pubertal status on radial diffusivity in clusters showing a main effect of age underlined that known differences in pubertal onset could be a driving factor for sexual dimorphism in diffusion parameters.

More recently, Bava et al. (2011) have shown that structural white matter development was generally greater in females than in males. They concluded that microstructural changes in adolescent females were mainly in the motor tracts, whereas the changes in adolescent males were relatively more focused in association and projection fibers. Herting et al. (2012) presented findings in a younger cohort of 77 adolescents from 10 to 16 years and with TBSS observed several white matter regions where males had lower MD than females. Other regions were identified where males had higher FA values than females.

Some (but not all) of these findings were consistent with those of Schmithorst et al. (2008), whose cohort had a much broader age range from 5 to 18 years. However, the results of Herting et al. (2012) disagreed with those of Asato et al. (2010), whose cohort had a larger age range (8–28 years cf. 10–16 years). Although differences in the age ranges studied may be a contributory factor, the exact reasons for these discrepancies remain unclear.

We have previously shown, using tractography, differences in developmental trajectory between males and females in a cohort of children from 8 to 16 years (Clayden et al., 2012). In this work, we further explored possible differences between the sexes using TBSS in the same cohort, which allowed us to probe changes in white matter development on a much finer spatial scale and to examine the spatial distribution of structural differences between males and females throughout the white matter. The use of TBSS also allowed us to investigate changes in white matter regions that do not belong to any of the tracts investigated in our previous study (Clayden et al., 2012).

Importantly, this cohort included children younger than those studied by Asato et al. (2010) and Herting et al. (2012), a group more closely representing the presumed onset of puberty (Dorn, 2006; Spear, 2000). The analysis focused on four measures derived from the diffusion tensor: FA, MD, λ_radial_, and λ_axial_. As a separate analysis, the mean of each measure was also calculated over the FA skeleton generated by TBSS and plotted against age to give further insight into white matter development over the studied age range.

## Materials and Methods

### Participants

The study cohort consisted of 64 healthy children (36 female) aged from 8 to 16.7 years. Of these, 11 data sets were rejected due to imaging artifacts and were excluded from the subsequent analysis, leaving 53 children aged from 8.0 to 16.7 years old (boys' age range 8.0–16.7, mean 10.8 ± 2.1; girls' age range 8.2–15.5, mean 11.7 ± 1.9), of which 30 were female.

Children were recruited by advertising in local schools or through relationships to children being scanned as part of a clinical study. A standard questionnaire (Child Behavior Checklist (http://www.aseba.org) rating socioemotional and behavioral status of each participant was completed by one of the parents accompanying the child. However, no children had to be excluded. Full-scale intelligence quotient (FSIQ) values were obtained for each participant using the Wechsler Intelligence Scale for Children, fourth UK edition (WISC-IV UK) (Wechsler, 2004). The FSIQ was 92–134 (mean 110.6 ± 9.9) for the boys and 88–137 (mean 115.0 ± 11.8) for the girls in the cohort. The study was approved by the local ethics committee and informed written parental consent was obtained.

### Imaging methods

Imaging data were acquired using a Siemens Avanto 1.5T clinical scanner (Siemens Healthcare, Erlangen, Germany) with a self-shielded gradient set with maximum gradient amplitude of 40 mT m^−1^ and standard 12 channel “birdcage” head coil.

Echo-planar diffusion-weighted images were acquired along 20 noncollinear gradient directions at *b* =1000 sec mm^−2^, with a single *b* = 0 image for normalization and number of excitations was set to two. This protocol was repeated thrice in a single scan session providing 126 acquisitions per slice, and the data merged without averaging. A voxel matrix of 96 × 96 was used and 45 contiguous axial slices acquired, each 2.5 mm thick, with a 240 × 240 mm field of view providing a final image resolution of 2.5 × 2.5 ×2.5 mm. Echo time was 89 msec, and repetition time was 6300 msec.

In addition, a T1-weighted 3D FLASH structural image was also acquired in the same session with 176 contiguous sagittal slices, a 256 × 224 mm field of view providing 1 × 1 × 1 mm resolution. The echo time was 4.9 msec, and repetition time was 11 msec. The overall scan time, including sequences not reported here, was about an hour.

### Image processing and statistical analysis

Preprocessing of the data involved removal of eddy current distortions with the “eddy_correct” tool and skull stripping of the brain volume (Smith, 2002) using FSL (www.fmrib.ox.ac.uk/fsl). The TBSS pipeline is described in detail by Smith et al., 2006. Briefly, the process involves registering all FA maps to a template in standard space before averaging the transformed images to create a mean FA map. The mean FA map is then eroded to form an FA skeleton, which is thresholded to remove regions of high variability. Finally, the FA data of each subject are projected onto each voxel of the FA skeleton. In this work, we use an FA threshold of 0.2. To improve alignment, a study-specific template was generated to which all the subjects were registered. A video clip of the aligned FA maps is provided in the supplementary information (Supplementary Data are available online at www.liebertpub.com/brain).

Statistical tests were performed using FSL's randomize tool with 5000 random permutations to ensure convergence. In addition to FA, changes in the MD, λ_axial_, and λ_radial_ were also investigated. Direct comparison of DT metrics between males and females was performed while controlling for age, FSIQ, and total brain volume. The age–gender interaction with each DT measure was also investigated, as well as correlations between age and each DT measure for both males and females, using FSIQ and total brain volume as a covariate in each test. TBSS results included threshold-free cluster enhancement (Smith and Nichols, 2009) and were corrected for multiple comparisons using familywise error corrections. The mean of each diffusion parameter over the FA skeleton was then calculated for each subject and plotted against age to show overall relationships between male and female developmental trajectories in the DT metrics. Plotting of the mean of each measure against age was performed using the ggplot2 package (Wickham, 2009) in R.

The effects of normal brain development and gender on white matter microstructure were investigated using an analysis of covariance (ANCOVA), implemented in the CAR package (Fox and Weisberg, 2011) in R. The mean of each diffusion parameter over the white matter skeleton was regressed against a linear model containing main effect terms for gender, age, and FSIQ, as well as interaction terms between all combinations of the main effects.

## Results

In all of the TBSS figures shown, the results are overlaid onto the MNI152 T1-weighted template with the TBSS FA skeleton highlighted in green. The axial slices shown are in the range z = 46 to z = 126 in 10 slice increments, the sagittal slices are x = 57, x = 90, and x = 123, and the coronal slices are y = 81, y = 107, and y = 134. Specific tracts were identified using the JHU ICBM-DTI-81 white matter labels atlas (Mori et al., 2008), available as part of the FSL toolkit.

### Female versus male

**FIG. 1.**
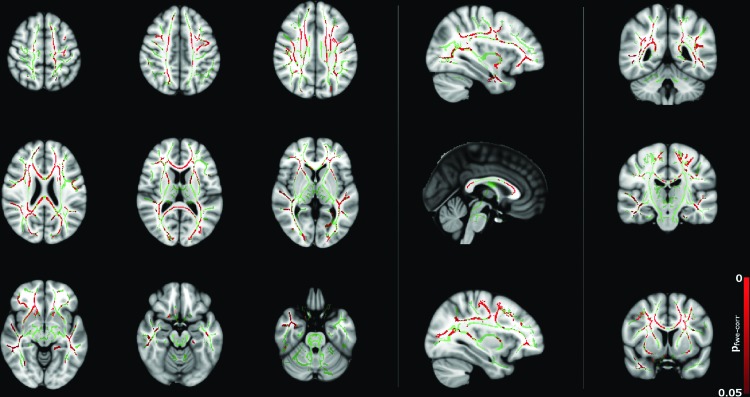
Group differences in mean diffusivity (MD) between male and female subjects (*p* < 0.05, corrected), corrected for age, total brain volume, and full-scale intelligence quotient (FSIQ). Red regions indicate a significantly higher MD in males than females. Color images available online at www.liebertpub.com/brain

Direct comparison of DT metrics between males and females is shown in Figure 1 and Supplementary Figures S1 and S2, overlaid onto the FSL MNI152 T1 template. MD was found to be greater in males than females across the majority of the white matter skeleton, as shown in Figure 1. These regions included but were not restricted to the corticospinal tracts bilaterally, tracts within the superior frontal gyrus bilaterally, internal and external capsules bilaterally, thalamic radiations, superior longitudinal fasciculi, corona radiata bilaterally, left cingulum corpus callosum, and tracts within the right inferior frontal gyrus. No regions were detected where MD was higher in females than in males.

Both λ_axial_ and λ_radial_ were also found to be higher in males than in females primarily within the corpus callosum for λ_axial_ as shown in Supplementary Figure S1 and in widespread white matter regions (similar to those for MD, with the addition of cerebral/cerebellar peduncles and the pontine crossing tract) for λ_radial_ as shown in Supplementary Figure S2. Similar to MD, no regions were found where either λ_axial_ or λ_radial_ was higher in females compared to males. No regions were found where FA was statistically significantly different in males compared to females.

**FIG. 2.**
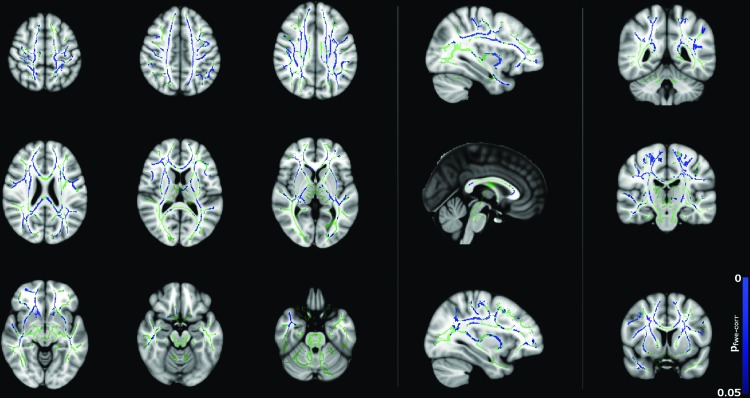
Age–gender interactions in MD between male and female subjects (*p* < 0.05, corrected), corrected for total brain volume and FSIQ. Blue regions indicate a significantly steeper negative slope in males than females. Color images available online at www.liebertpub.com/brain

**FIG. 3.**
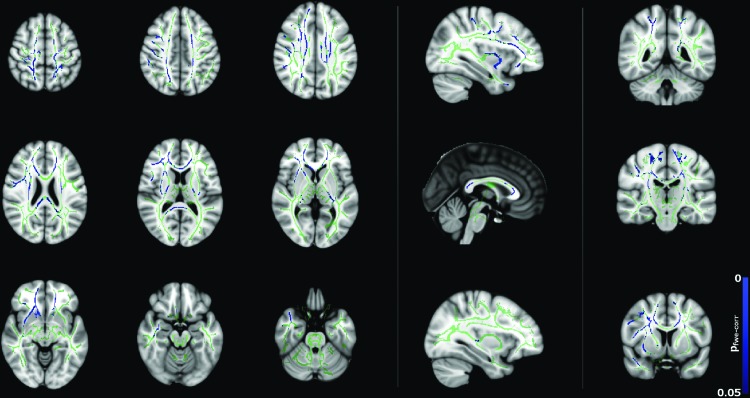
Age–gender interactions in radial diffusivity (λ_radial_) between male and female subjects (*p* < 0.05, corrected), corrected for total brain volume and FSIQ. Blue regions indicate a significantly steeper negative slope in males than females. Color images available online at www.liebertpub.com/brain

Figures 2 and 3 show significant (*p* < 0.05) age–gender interactions for MD and λ_radial_, respectively. The age–gender interaction for MD (Fig. 2) reveals a more negative slope for males than females, specifically in the corpus callosum, external/internal capsules bilaterally, corona radiata bilaterally, superior longitudinal fasciculus bilaterally, sagittal stratum bilaterally, and left posterior thalamic radiation. The slope for λ_radial_ (Fig. 3) is also negative, but reaches significance in far fewer regions (corpus callosum, superior/anterior corona radiata bilaterally, posterior limb of the internal capsule bilaterally, left retrolenticular portion of the internal capsule and right external capsule) and is more pronounced in the superior portion of the brain.

**FIG. 4.**
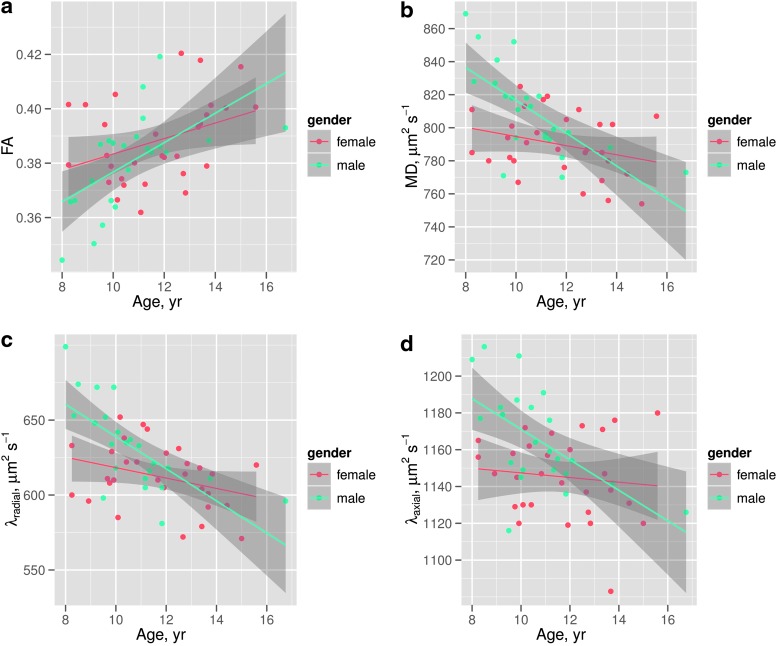
Correlation between the mean value across the fractional anisotropy (FA) skeleton for **(a)** FA, **(b)** MD, **(c)** λ_radial_, and **(d)** axial diffusivity (λ_axial_) with age for both males (blue) and females (red). Shaded area refers to the standard error on the fit. Color images available online at www.liebertpub.com/brain

Plots for each of the tensor measures, averaged over the FA skeleton, against age for each participant, are shown in Figure 4. The plots show an increase in (a) FA and decreases in (b) MD, (c) λ_radial_, (d) λ_axial_ for the male subjects with age, but either weak or no correlation with age for the female subjects. The plot of FA against age (Fig. 4a) suggests that the FA for male subjects is lower than for the female subjects in the youngest portion of the cohort, but the difference between genders decreases with age and is negligible by the age of around 10–14 years old. The other plots show similar trends for the diffusivity metrics, where the diffusivity is higher for the youngest male subjects in the cohort but older male subjects have more similar diffusivities to female subjects (Fig. 4b–d). The plot of λ_axial_ against age (Fig. 4d) shows a weaker correlation.

The results of the ANCOVA are shown in Table 1. The analysis shows a highly significant main effect of age in all diffusion metrics. In addition to this, there is a significant main effect of brain volume on the diffusivity metrics. There is evidence that these effects differ by gender, as indicated by the age–gender and gender–brain volume interactions. A gender–FSIQ interaction on FA, MD, and λ_radial_ was also observed. Terms that are not significant have been omitted from the table.

**Table 1.** Analysis of Covariance of Diffusion Metrics, Averaged Over the TBSS Skeleton, Against Age, Gender, Full-Scale Intelligence Quotient, and Total Brain Volume

	*FA*	*MD*	***λ_axial_***	***λ_radial_***
Age	F (1,37) = 18.26	F (1,37) = 28.71	F (1,37) = 12.11	F (1,37) = 28.61
	*p* < 0.001^a^	p < 0.001^a^	*p* = 0.001^b^	*p* < 0.001^a^
Brain volume	F (1,37) = 0.06	F (1,37) = 9.77	F (1,37) = 14.11	F (1,37) = 5.07
	*p* = 0.807	*p* = 0.003^b^	*p* < 0.001^a^	*p* = 0.030^c^
Age–Gender	F (1,37) = 1.19	F (1,37) = 6.40	F (1,37) = 4.47	F (1,37) = 5.21
	*p* = 0.283	*p* = 0.016^c^	*p* = 0.041^c^	*p* = 0.028^c^
Gender–FSIQ	F (1,37) = 4.33	F (1,37) = 4.58	F (1,37) = 1.13	F (1,37) = 5.41
	*p* = 0.044^c^	*p* = 0.039^c^	*p* = 0.295	*p* = 0.026^c^
Gender–Brain volume	F (1,37) = 4.90	F (1,37) = 3.17	F (1,37) = 0.31	F (1,37) = 4.47
	*p* = 0.033^c^	*p* = 0.083	*p* = 0.578	*p* = 0.041^c^
FSIQ–Brain volume	F (1,37) = 3.01	F (1,37) = 1.22	F (1,37) = 0.001	F (1,37) = 2.28
	*p* = 0.091	*p* = 0.276	*p* = 0.971	*p* = 0.139
Age–gender–brain volume	F (1,37) = 1.81	F (1,37) = 0.44	F (1,37) = 3.87	F (1,37) = 0.02
	*p* = 0.187	*p* = 0.511	*p* = 0.057	*p* = 0.897

^a^ Significant at the *p* < 0.001 level.

^b^ Significant at the *p* < 0.01 level.

^c^ Significant at the *p* < 0.05 level.

Close to significance (*p* < 0.1).

Terms that do not reach significance for any of the diffusion metrics have been omitted.

λaxial, axial diffusivity; λradial, radial diffusivity; FA, fractional anisotropy; FSIQ, full-scale intelligence quotient; MD, mean diffusivity; TBSS, tract-based spatial statistics.

### Males

**FIG. 5.**
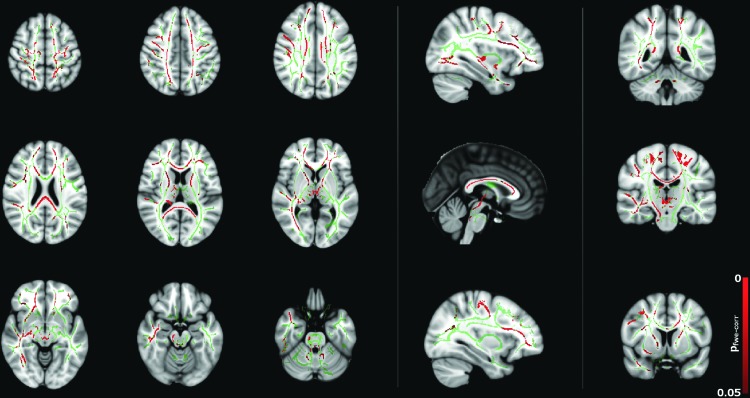
Correlation between FA and age for the male subjects (*p* < 0.05, corrected), corrected for FSIQ and total brain volume. Red regions indicate a significant increase in FA. Color images available online at www.liebertpub.com/brain

Figure 5 shows TBSS results (correlation between FA and age) for the male subgroup. The areas highlighted in red indicate regions where there is a significant (*p* < 0.05) positive correlation between FA and age in the male subjects. The FA increases occur throughout the brain, which is in agreement with findings on child–adolescent development (Clayden et al., 2012; Giorgio et al., 2008; Lebel et al., 2008;). Specifically, there is a positive correlation between age and FA in the corpus callosum, corona radiata bilaterally, anterior limb of the internal capsule bilaterally, right posterior limb of the internal capsule, right posterior thalamic radiation, right cerebral peduncle, and right sagittal stratum. No significant negative correlations between FA and age were observed.

**FIG. 6.**
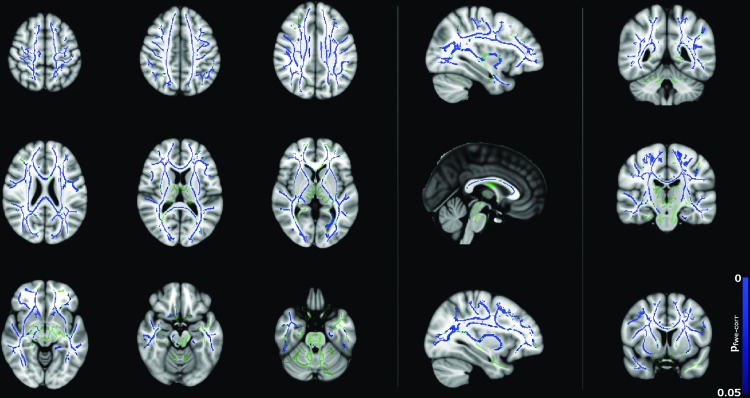
Correlation between MD and age for male subjects (*p* < 0.05, corrected), corrected for FSIQ and total brain volume. Blue regions indicate a significant decrease in MD. Color images available online at www.liebertpub.com/brain

Figure 6 shows TBSS results of regions of significant (*p* < 0.05) negative correlation between MD and age for the male subjects. The analysis suggests there is a widespread decrease in MD across the skeleton, in broadly the same areas where FA increases. There are, however, several regions where there is a significant negative correlation between MD and age that are not significant for FA. These are the cingulum bilaterally, external capsules bilaterally, superior longitudinal fasciculus bilaterally, sagittal stratum bilaterally, and left thalamic radiation.

**FIG. 7.**
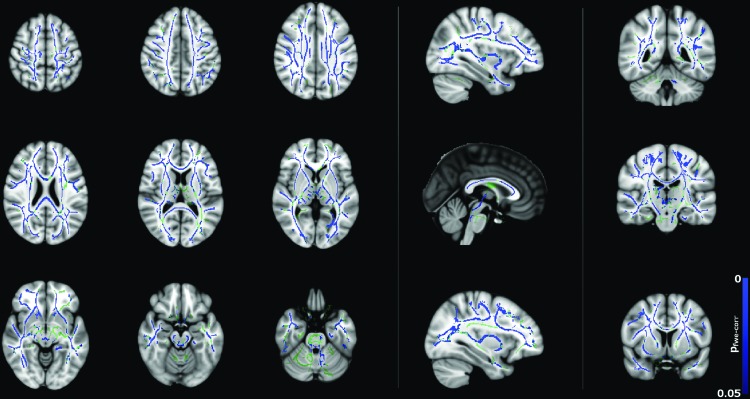
Correlation between λ_radial_ and age for the male subjects (*p* < 0.05, corrected), corrected for FSIQ and total brain volume. Blue regions indicate a significant decrease in λ_radial_. Color images available online at www.liebertpub.com/brain

**FIG. 8.**
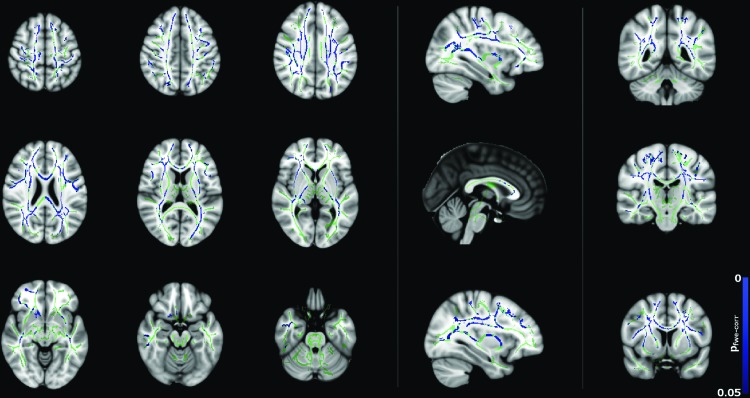
Correlation between λ_axial_ and age for the male subjects (*p* < 0.05, corrected), corrected for FSIQ and total brain volume. Blue regions indicate a significant decrease in λ_axial_. Color images available online at www.liebertpub.com/brain

Negative correlations between λ_radial_ with age for males are shown in Figure 7. There are widespread significant (*p* < 0.05) changes in λ_radial_ with age, whereas far fewer significant changes in λ_axial_ were observed for males (Fig. 8). This may indicate that the changes observed for FA and MD are mainly driven by reductions in λ_radial_. However, λ_radial_ is the mean of the two minor eigenvalues of the tensor as opposed to λ_axial_, which uses only the principal eigenvalue. Therefore, the difference may be, in part, due to the fact that λ_radial_ is less noisy than λ_axial_.

### Females

**FIG. 9.**
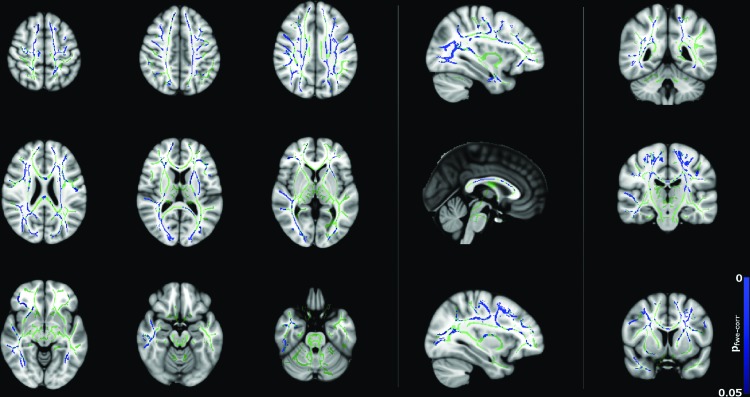
Correlation between MD and age for female subjects (*p* < 0.05, corrected), corrected for FSIQ and total brain volume. Blue regions indicate a significant decrease in MD. Color images available online at www.liebertpub.com/brain

**FIG. 10.**
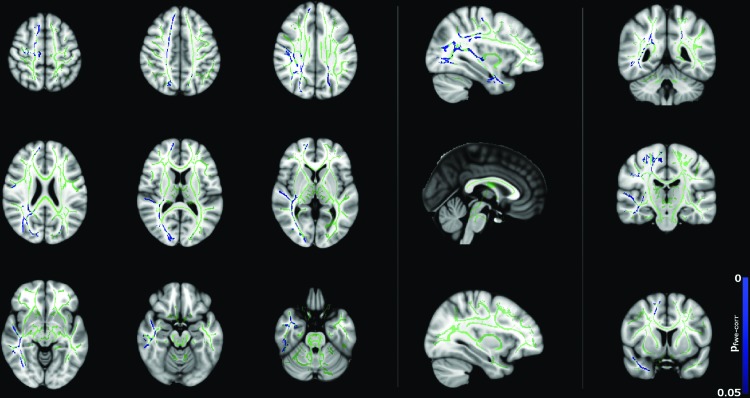
Correlation between λ_radial_ and age for the female subjects (*p* < 0.05, corrected), corrected for FSIQ and total brain volume. Blue regions indicate a significant decrease in λ_radial_. Color images available online at www.liebertpub.com/brain

Figure 9 shows negative correlation between MD and age for the female subjects, although there are fewer significant voxels compared to the male cohort (Fig. 6). Regions include the corpus callosum, corona radiata bilaterally, superior longitudinal fasciculus bilaterally, thalamic radiations bilaterally, the left external/internal capsules, and the right retrolenticular portion of the internal capsule. In contrast to the widespread correlation between λ_radial_ with age for males (Fig. 7), correlation between λ_radial_ with age for females (Fig. 10) is far less widespread than for males (only the left posterior corona radiata, right corona radiata, right superior longitudinal fasciculus, right thalamic radiation, and right retrolenticular portion of the internal capsule) and there are no significant correlations between λ_axial_ and age in this group.

The TBSS analysis of the female subgroup showed no significant correlation of FA with age after correction for multiple comparisons.

### 8 to 13 age range

To account for potential confounds caused by a differing distribution of ages between the genders, we repeated the TBSS analyses using a subset of data in the 8–13-year age range, which has a similar coverage of age range for both genders. Results from the TBSS analysis of the 8–13-year olds are also widespread across the TBSS skeleton and also show a clear age–gender interaction (see Supplementary Figs. S3–S12 in supplementary information). In addition to the age–gender interactions with diffusivity measures, there is also a widespread age–gender interaction with FA across the TBSS skeleton in the 8–13 range (Supplementary Fig. S6). In the 8–16 range, the age–gender interaction with FA is not significant, but does reach close to significance (*p* < 0.1) in a subset of the regions shown in the 8–13 group.

## Discussion and Conclusions

The primary finding in this study is that there are differences in structural development *throughout* the white matter in males and females between the ages of 8 and 16 years. Furthermore, this difference prevails when corrected for FSIQ and total brain volume. These findings extend those of Asato et al. (2010) that described differences in λ_radial_ in specific brain regions with higher values in males compared with females using a significance threshold of *p* < 0.001 (corrected). FSIQ has been shown to be correlated with diffusion parameters in development (Schmithorst et al., 2010), and we have recently shown that factors of white matter integrity based on FA and MD in 14 specific tracts independently predict FSIQ (Clayden et al., 2012). These factors were primarily linked to the corpus callosum, but were also influenced by the left-sided inferior longitudinal and arcuate fasciculi.

The approach taken in this study was therefore to control for possible differences in FSIQ between males and females in the 8–16 years age range to isolate gender differences in the trajectory of diffusion tensor metrics. In addition to the study by Asato et al. (2010), differences were observed in the trajectory of MD, λ_axial_, λ_radial_, and FA between males and females. These trajectories differed such that males had lower FA and higher MD, λ_radial_, and λ_axial_, particularly between 8 and 9 years of age compared to females, with a steeper slope of correlation. However, the diffusion parameters for the males appeared to be indistinguishable to the corresponding parameters in females in the 10 to 14 years age range, as shown in Figure 4.

The trends in Figure 4a, b of the present study broadly agree with those shown in Figures 2 and 3 of Clayden et al. (2012). Specifically, both studies show the trend of increasing FA and decreasing MD with age for boys but not girls in the 8–16 age range. This difference in slope was reflected in the TBSS age–gender interactions, which showed widespread age–gender effects for FA and MD and—to a lesser extent—λ_radial_. In the ANCOVA, age–gender interactions were significant for MD, λ_axial_, and λ_radial_ as shown in Table 1. MD, λ_axial_, and λ_radial_ also showed a significant effect of brain volume, which is consistent with previous studies (e.g., Clayden et al., 2012), while in FA, this effect was gender dependent. Interactions between FSIQ and gender were significant for FA, MD, and λ_radial_.

Correlations between diffusion tensor metrics and age for males were observed throughout white matter, as can be seen in Figures 5–8. These correlations were less apparent in the TBSS analysis for the female subjects (Figs. 9, 10) due to the shallow slope of correlation in diffusion tensor metrics observed in females. These differences were further illustrated when DT metrics were directly compared between males and females using TBSS. Greater MD values in males compared to females were found in widespread regions across the FA skeleton as shown in Figure 1. Similar findings were observed but in fewer voxels within the skeleton for λ_axial_ and λ_radial_, as shown in Supplementary Figures S2 and S3.

The precise mechanisms for these observed differences in structural trajectory remain to be elucidated. However, it has been suggested that the onset of puberty may play an important role (Asato et al., 2010; Blakemore et al., 2010; Herting et al., 2012). During childhood, several hormonal changes occur and these include adrenarche, gonadarche, and activation of the growth axis. Adrenarche is characterized by activation of the hypothalamic–pituitary–adrenal axis with a typical onset between 6 and 9 years in females and 7 and 10 years in males. Adrenarche is followed by gonadarche, which is characterized by activation of the hypothalamic–pituitary–gonadal axis resulting in the attainment of reproductive competence.

Typical onset of this process occurs at a mean age of 11 years (range 8–14 years) in females and at a mean age of 12 years (range 9–15 years) in males (Spear, 2000; Dorn, 2006), which is covered well by our sample. Activation of the growth axis results in a linear growth spurt around the age of 12 in females and age 14 in males (Marshall and Tanner, 1969, 1970). These processes, particularly adrenarche, occur during the period when the greatest differences in structural trajectory were observed between males and females and, therefore, represent a possible candidate mechanism driving the observed sexually dimorphic structural trajectory.

A recent study in 10–16-year olds also investigated sex differences in white matter structure using TBSS together with the effects of puberty (Herting et al., 2012). In contrast to the present study, they found a number of clusters where FA in males was found to be higher than that in females. The differences between this pattern and the results in the present study may be due to differences in the age ranges studied, which were 8–16 years in the present study and 10–16 years in the study of Herting et al. (2012). One would envisage that, therefore, activation of the growth axis would be more prevalent in this latter cohort, particularly in children above the age of 12. However, in contrast to the study of Herting et al. (2012), Bava et al. (2011) reported similar findings to those reported here, namely greater MD and λ_axial_ and lower FA in males compared to females using TBSS, but in much smaller regions than demonstrated in the present study. The age range studied was from 12 to 14 years with a mean age of the cohort of 13.4 years, which is close to that of the cohort studied by Herting et al. However, Bava et al. did detect two clusters in the left and right corticospinal tract, respectively, where λ_axial_ was greater in females than males.

Using magnetization transfer imaging, which measures the degree of macromolecular content in brain tissue and is thought to be an indirect measure of myelin content, Perrin et al. (2008, 2009) observed, in a cohort spanning 12 to 18 years, a decrease in the magnetization transfer ratio in males with age but no relationship with age in females. They also found a more rapid increase in white matter volume in males with age compared to females (Perrin et al., 2008, 2009).

Herting et al. (2012) provided further evidence for the influence of puberty on structural white matter trajectories between males and females, demonstrating correlation between FA and pubertal development scale (PDS) in the white matter of the insular gyrus and a gender by PDS interaction for FA in the superior frontal gyrus. In addition, FA was positively correlated with testosterone levels in males in clusters in the left superior frontal gyrus, left corpus callosum, right posterior limb of the internal capsule, and left angular gyrus white matter with a negative correlation with FA in a cluster in the middle cerebellar peduncle. In females, FA positively correlated with testosterone in a cluster in the precentral gyrus white matter. Furthermore, in males, FA was positively correlated with estradiol in clusters in the right superior frontal gyrus, right precuneus, and the hippocampal part of the cingulum bilaterally. In females, FA was negatively correlated with estradiol in clusters in the right superior longitudinal fasciculus and left angular gyrus. These findings provide further confirmatory evidence for the influential role of puberty on sexually dimorphic structural white matter trajectories.

In the present study, more widespread differences in white matter DT parameters were observed between males and females than in the studies of Herting et al. (2012), Asato et al. (2010), and Schmithorst et al. (2008). However, in common with the study of Asato et al. (2010) and Bava et al. (2011), structural white matter development was generally greater in females than in males. Given the cascade of pubertal and endocrine events in children from around the age of 6 years onward and their differential onset and trajectory between the genders, it appears highly likely that these processes play an important role in structural white matter development.

These many events underline the importance of the exact age range studied, as different processes are more relevant at different ages. Ideally, further studies could be performed within tight age bands, for example, 1-year bands that would help to simplify the interpretation of results. In addition to this, the collection of information on pubertal status and sex steroids in the manner described by Herting et al. (2012) would help to elucidate the role of these processes on structural white matter development, a role which presumably changes across the range of ages from 6 to 18 years.

Results of voxel-based analyses of diffusion tensor data can be affected by several factors, including poor alignment of data (Van Hecke et al., 2009). The data in this analysis were checked at each step to ensure that the findings were not adversely influenced by any of these factors. In particular, the raw diffusion-weighted data and tensor reconstructions were checked visually for artifacts, and the registration was checked by playing a movie clip of FA maps to ensure close alignment before statistical analysis.

A limitation of the present work is the absence of measurements of pubertal stage. It is known that the timing and activity of pubertal hormones are significantly different between the genders (Marshall and Tanner, 1969, 1970), and brain development appears to be more closely correlated with the pubertal stage in girls than in boys (Blanton et al., 2012). Future work will therefore involve investigating how changes in white matter microstructure relate to Tanner stage or measures of pubertal hormones to further understand the differences in the developmental trajectories.

In summary, this study provides evidence for *widespread* sexually dimorphic trajectories in structural white matter development in children from 8 to 16 years with more ongoing development indicated by higher FA and lower MD, λ_axial_, and λ_radial_ in females compared to males. This difference in diffusion parameters appeared to be largest in the 8–9 year age range, with male and female trajectories tending to converge between 10 and 14 years of age. Further studies are now warranted to determine the role of pubertal hormones on the observed differences, particularly in 8–9-year-old children.

## Supplementary Material

Supplemental data

Supplemental data

Supplemental data

Supplemental data

Supplemental data
