# Supplementary material for: Sexual Dimorphism in White Matter Developmental Trajectories Using Tract-Based Spatial Statistics
Source: Brain Connect. 2016 Feb 1;6(1):37–47. doi: 10.1089/brain.2015.0340 (PMC4744889; doi:10.1089/brain.2015.0340)
Supplement: Supplemental data [file Supp_Figure1.pdf]

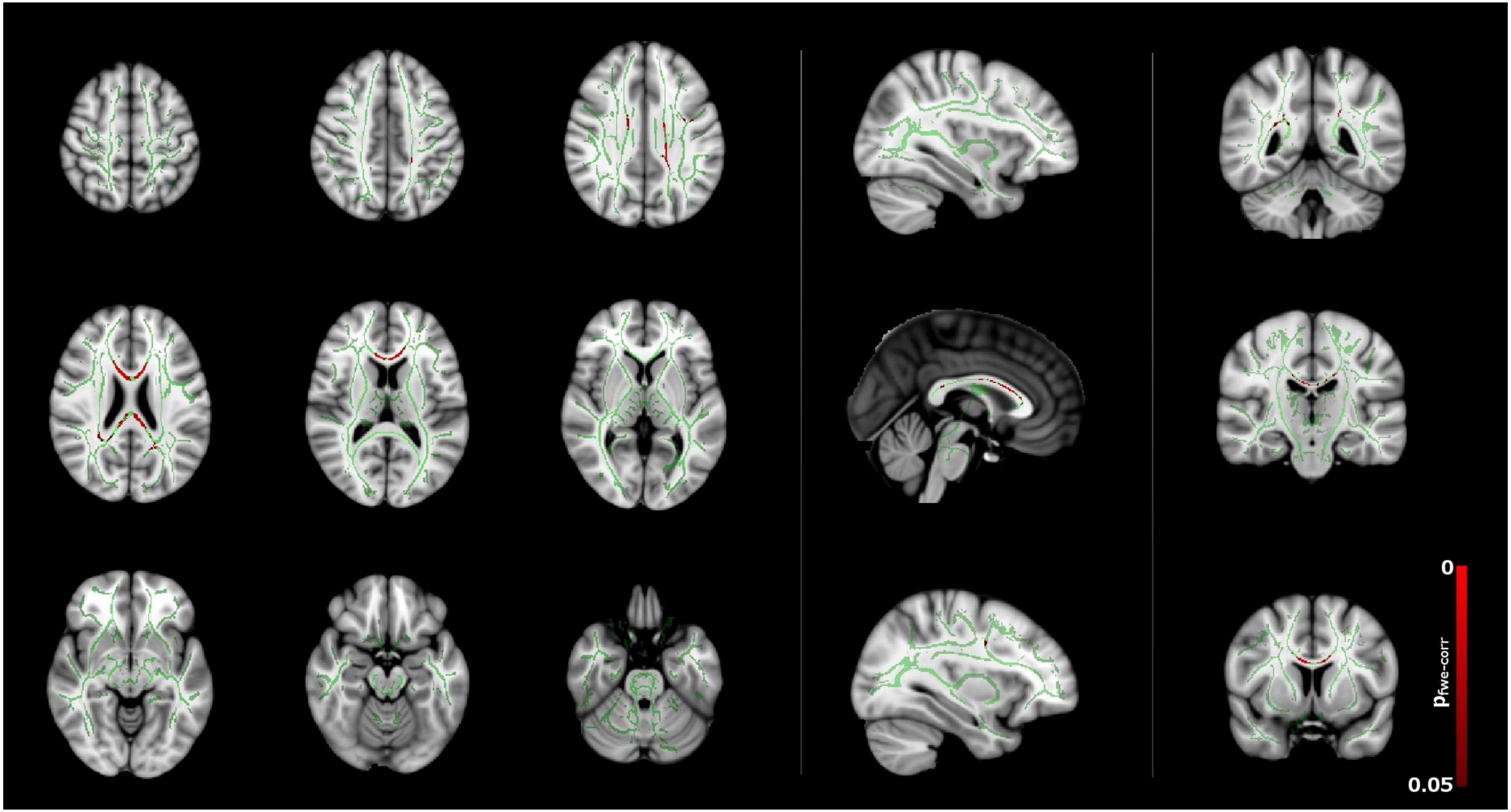

Supplementary Figure 1: Group differences in λ_{axial} between male and female subjects ($p<0.05$, corrected), corrected for age, total brain volume and FSIQ. Red regions indicate a significantly higher λ_{axial} in males than females.
