# Supplementary material for: Sexual Dimorphism in White Matter Developmental Trajectories Using Tract-Based Spatial Statistics
Source: Brain Connect. 2016 Feb 1;6(1):37–47. doi: 10.1089/brain.2015.0340 (PMC4744889; doi:10.1089/brain.2015.0340)
Supplement: Supplemental data [file Supp_Figure3-12.pdf]

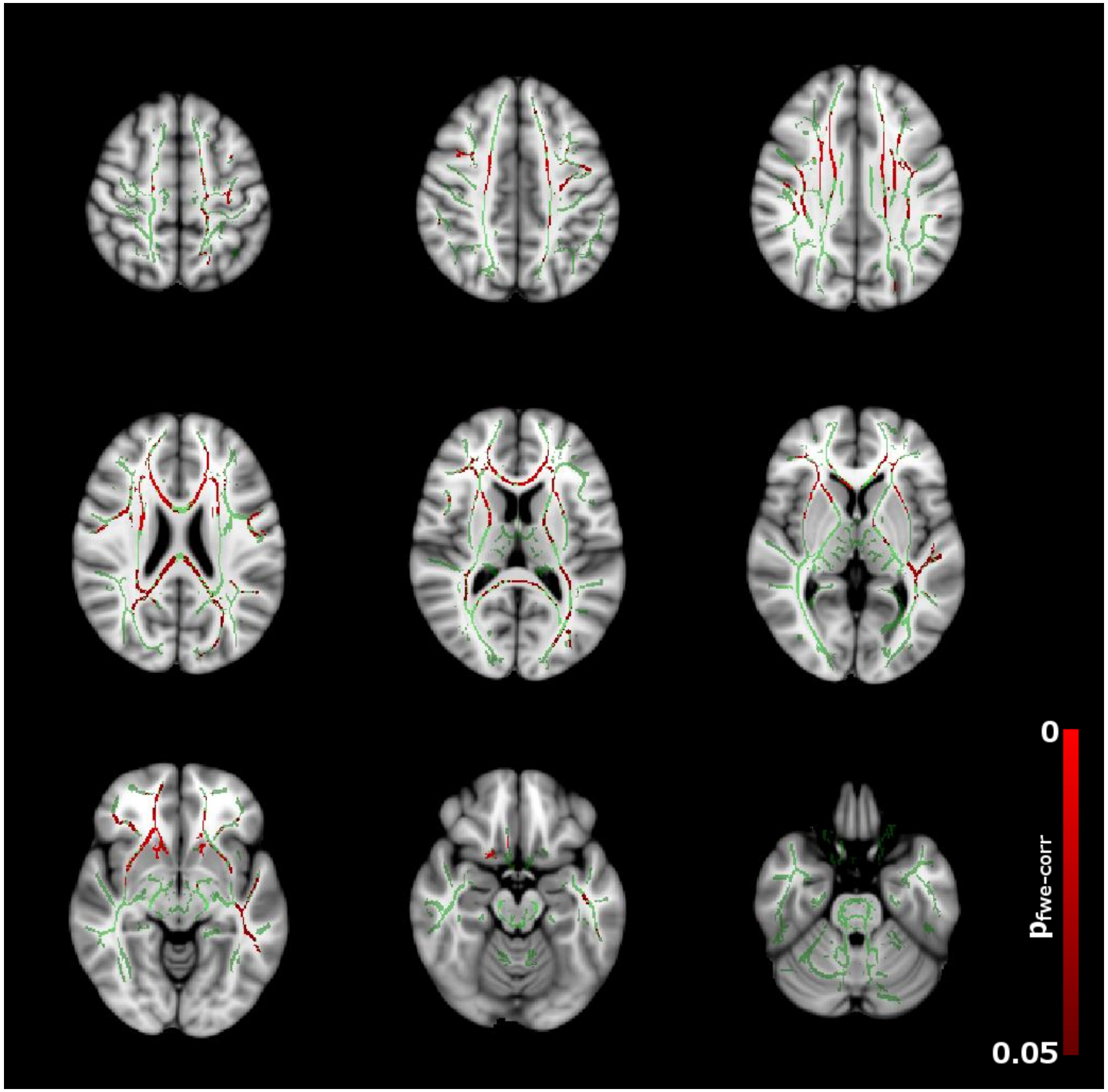

Supplementary Figure 3: Group differences in MD between male and female subjects (p<0.05, corrected) in the 8-13 age range, corrected for age, total brain volume and FSIQ. Red regions indicate a significantly higher MD in males than females. Corresponds to figure 1 in the main text (8-16 age range).

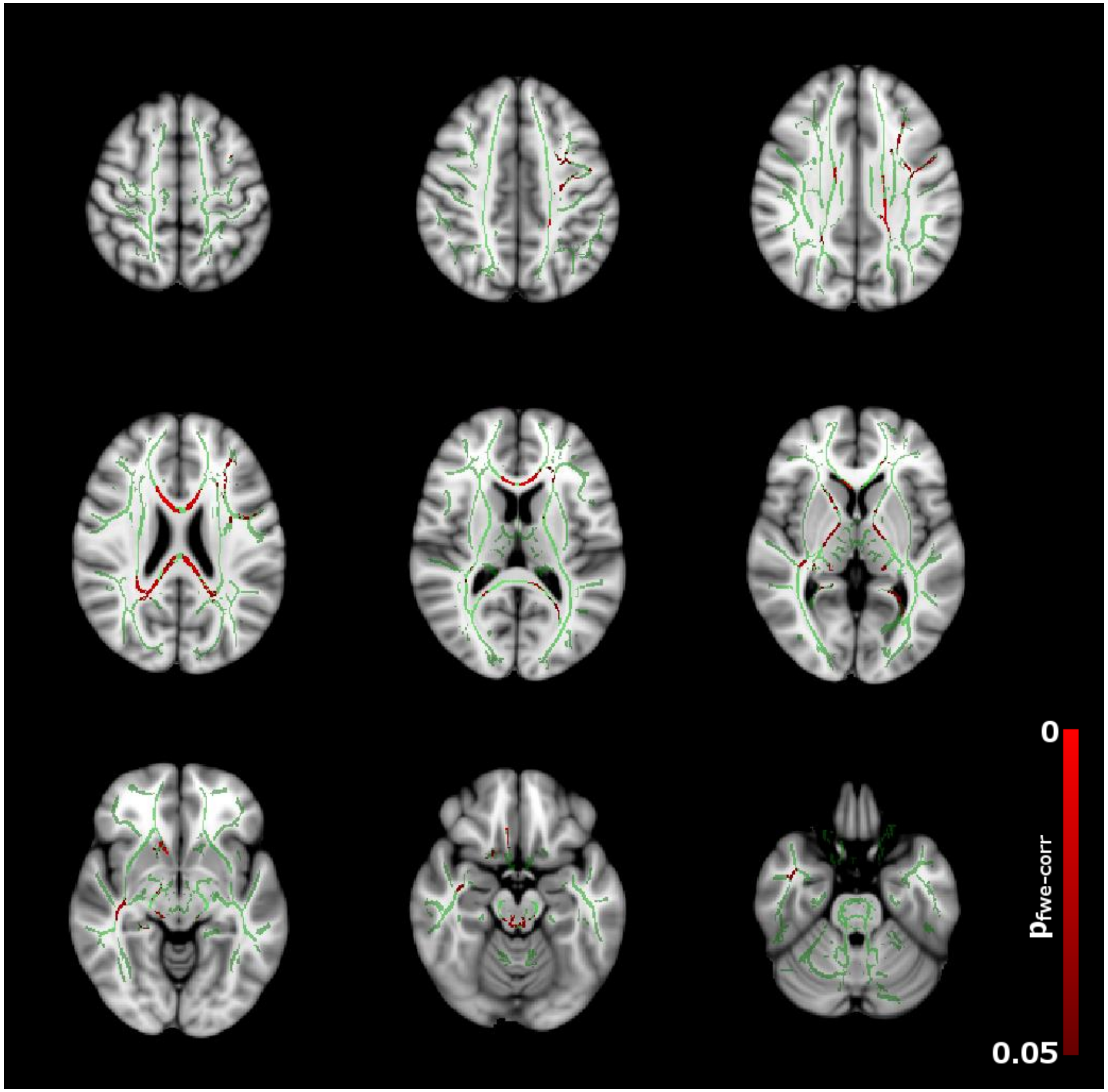

Supplementary Figure 4: Group differences in λ_{axial} between male and female subjects ($p<0.05$, corrected) in the 8-13 age range, corrected for age, total brain volume and FSIQ. Red regions indicate a significantly higher λ_{axial} in males than females. Corresponds to figure 2 in the main text (8-16 age range).

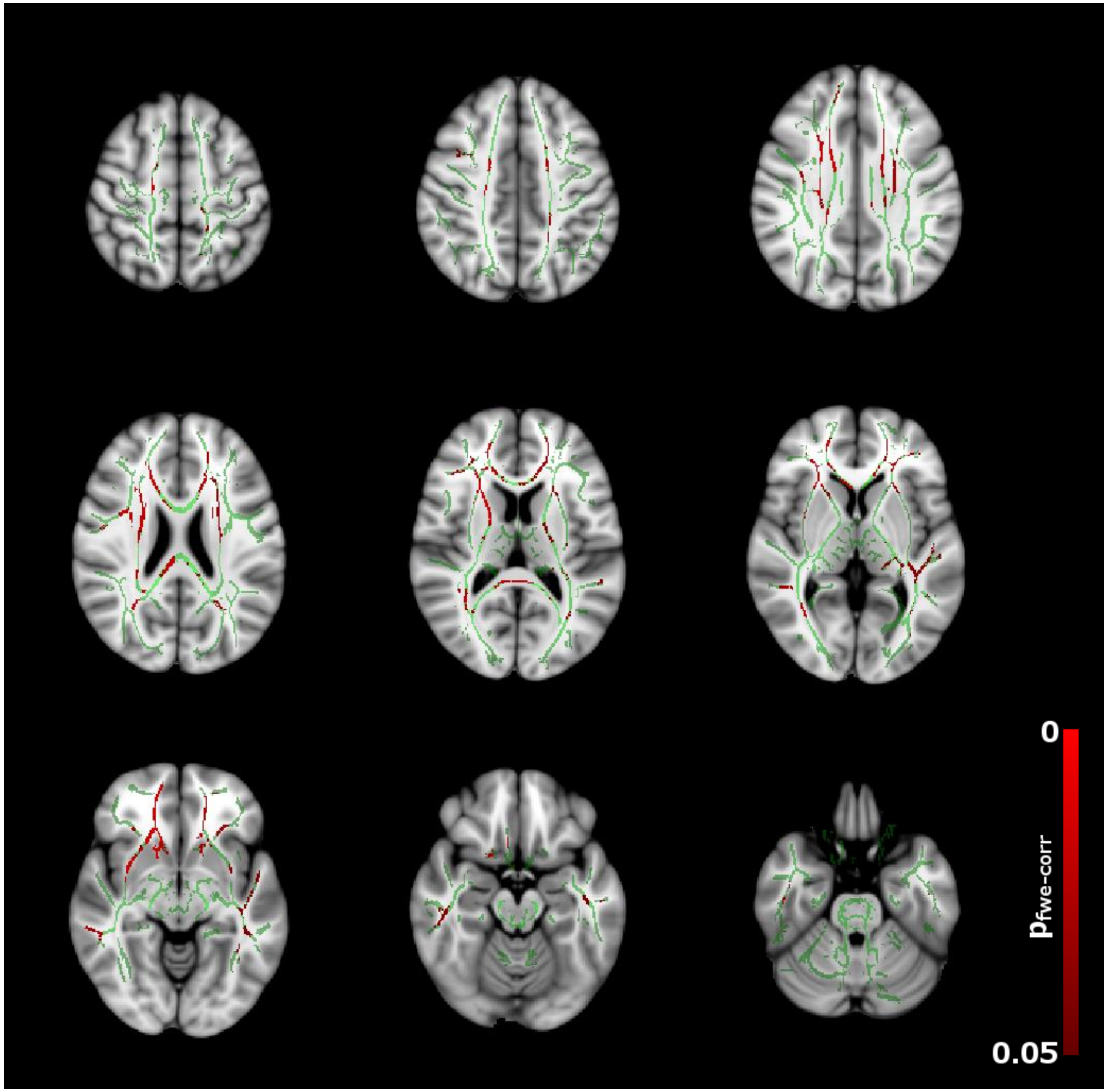

Supplementary Figure 5: Group differences in λ_{radial} between male and female subjects ($p<0.05$, corrected) in the 8-13 age range, corrected for age, total brain volume and FSIQ. Red regions indicate a significantly higher λ_{radial} in males than females. Corresponds to figure 3 in the main text (8-16 age range).

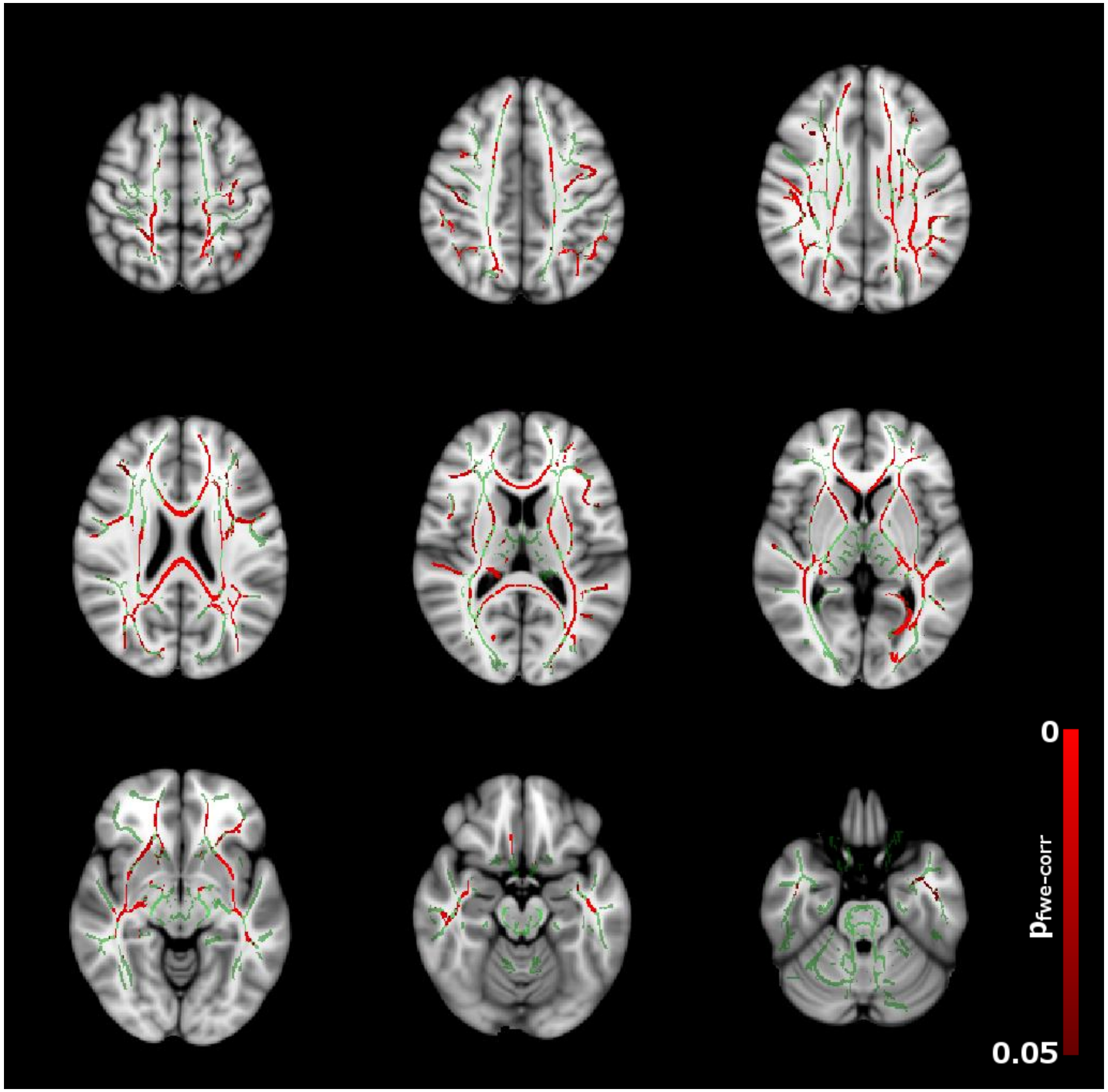

Supplementary Figure 6: Age-gender interactions in FA between male and female subjects ($p<0.05$, corrected) in the 8-13 age range, corrected for total brain volume and FSIQ. Red regions indicate a significantly steeper slope in males than females. No significant difference was seen in the 8-16 age range, although several regions were close to significance ($p<0.1$)

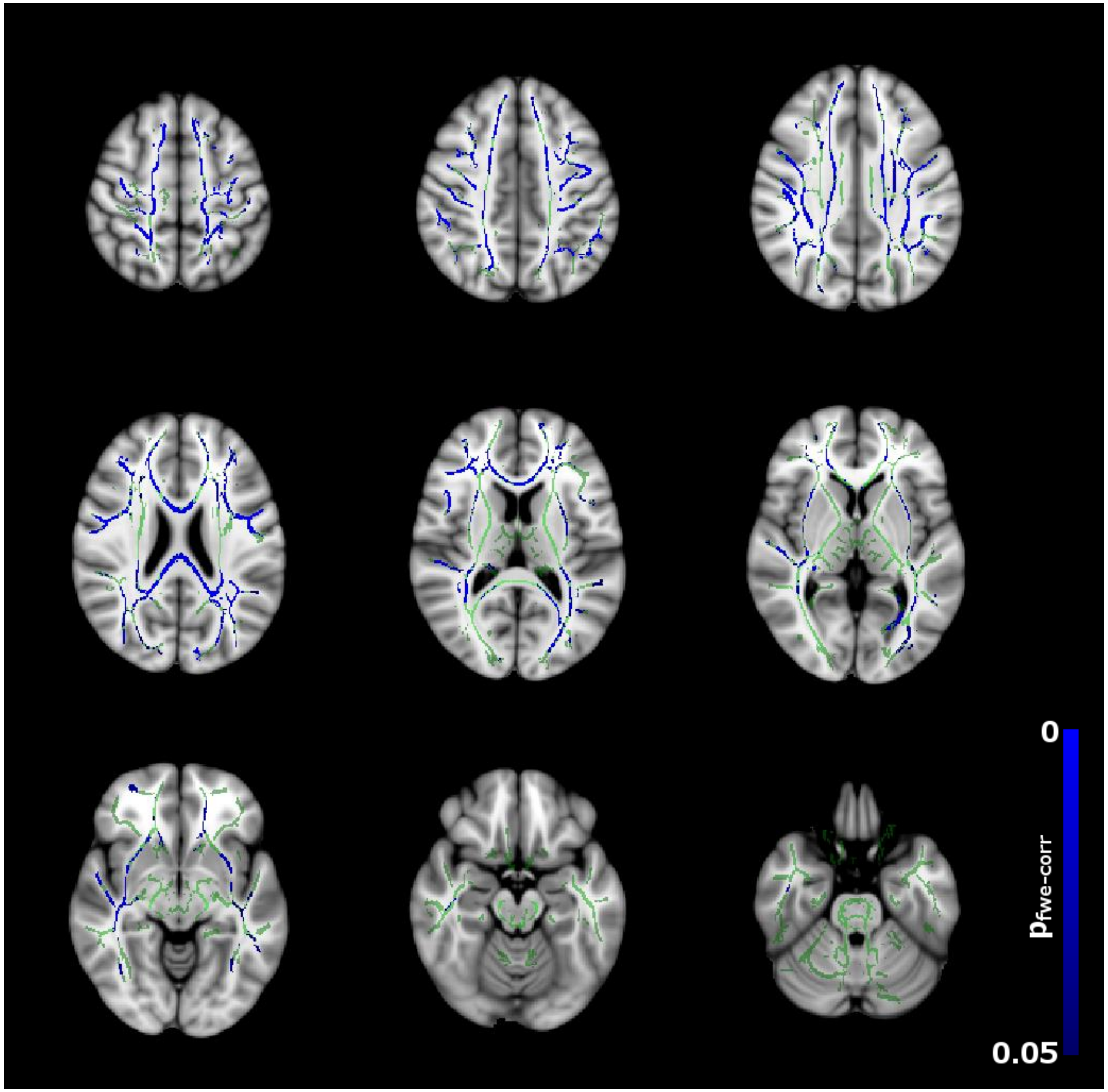

Supplementary Figure 7: Age-gender interactions in MD between male and female subjects ($p<0.05$, corrected) in the 8-13 age range, corrected for total brain volume and FSIQ. Blue regions indicate a significantly steeper negative slope in males than females. Corresponds to figure 4 in the main text (8-16 age range).

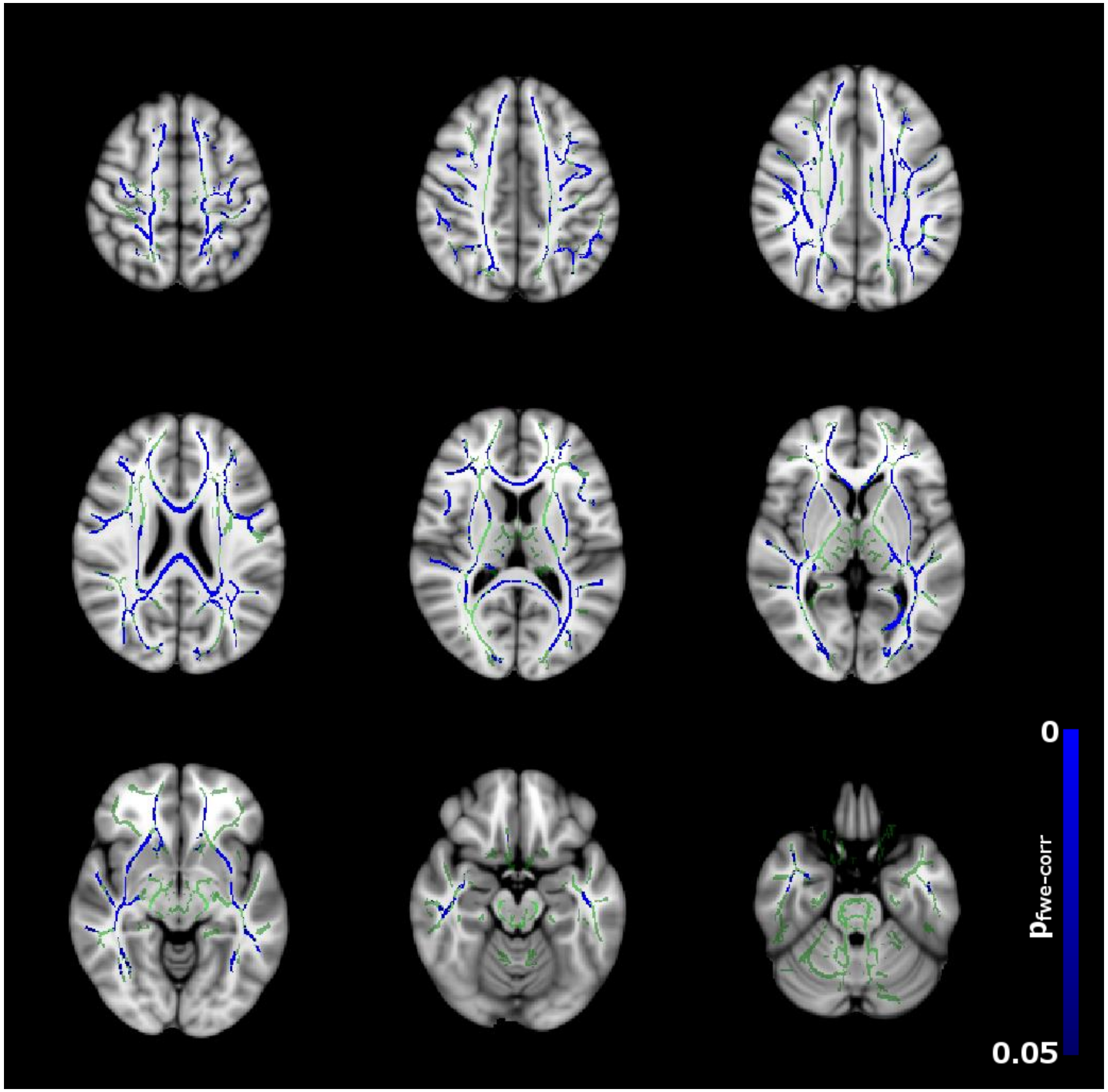

Supplementary Figure 8: Age-gender interactions in λ_{radial} between male and female subjects ($p<0.05$, corrected) in the 8-13 age range, corrected for total brain volume and FSIQ. Blue regions indicate a significantly steeper negative slope in males than females. Corresponds to figure 5 in the main text (8-16 age range).

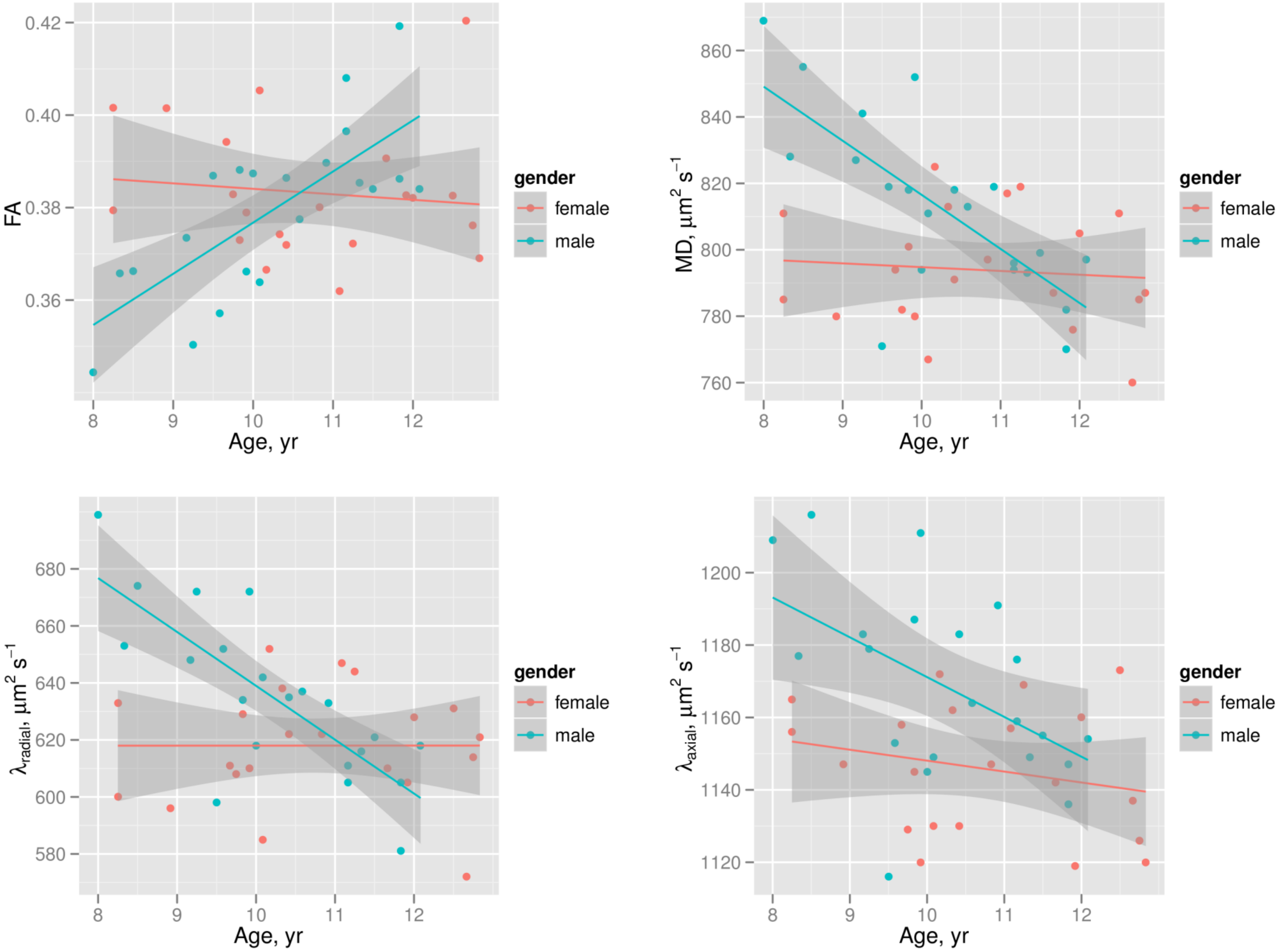

Supplementary Figure 9: Correlation between the mean value across the white matter skeleton for a) FA, b) MD, c) λ_{radial}, d) λ_{axial} with age for both males (blue) and females (red). Shaded area refers to the standard error on the fit. Corresponds to figure 6 in the main text (8-16 age range).

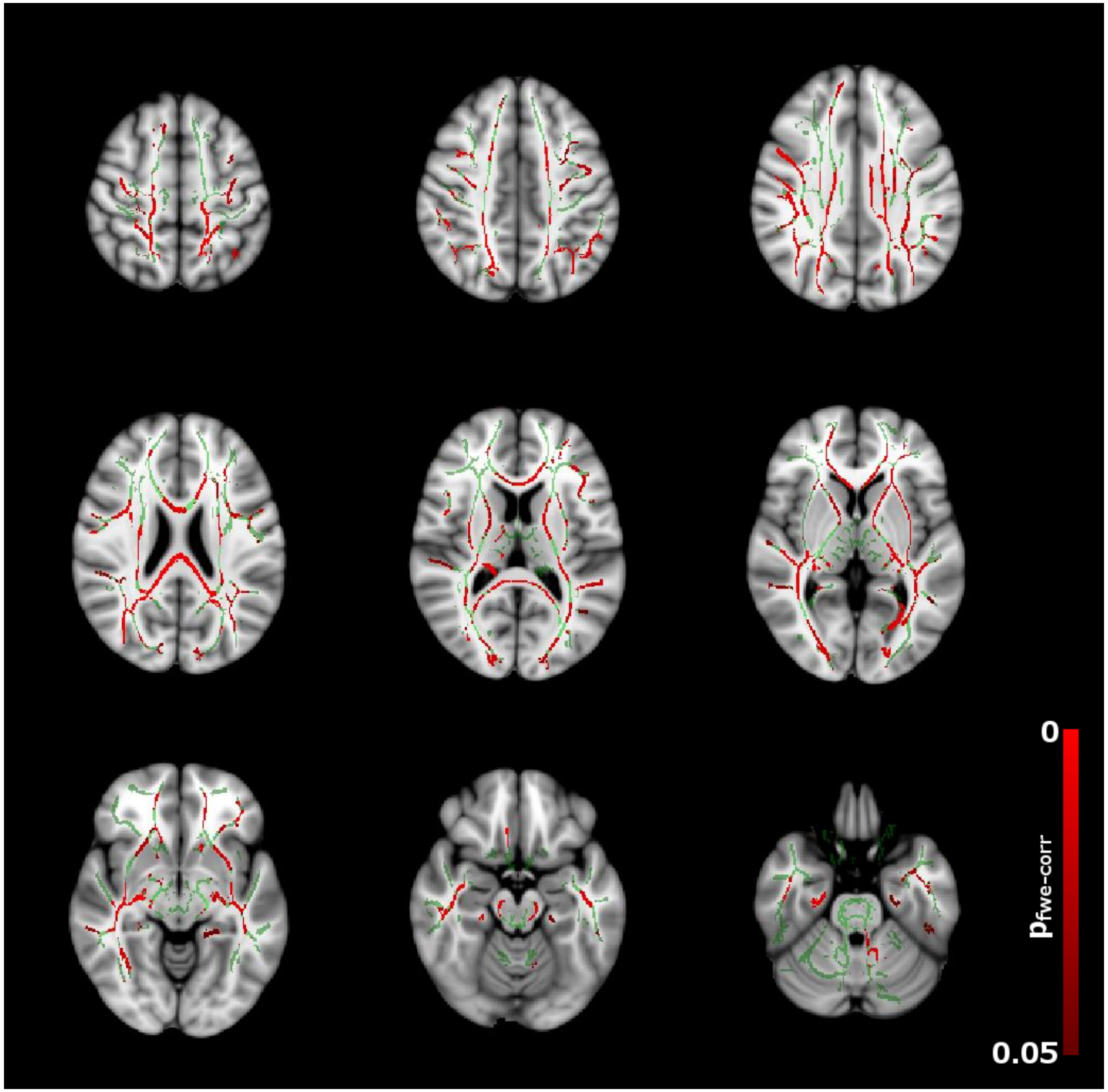

Supplementary Figure 10: Correlation between FA and age for the male subjects ($p<0.05$, corrected) in the 8-13 age range, corrected for FSIQ and total brain volume. Red regions indicate a significant increase in FA. Corresponds to figure 7 in the main text (8-16 age range).

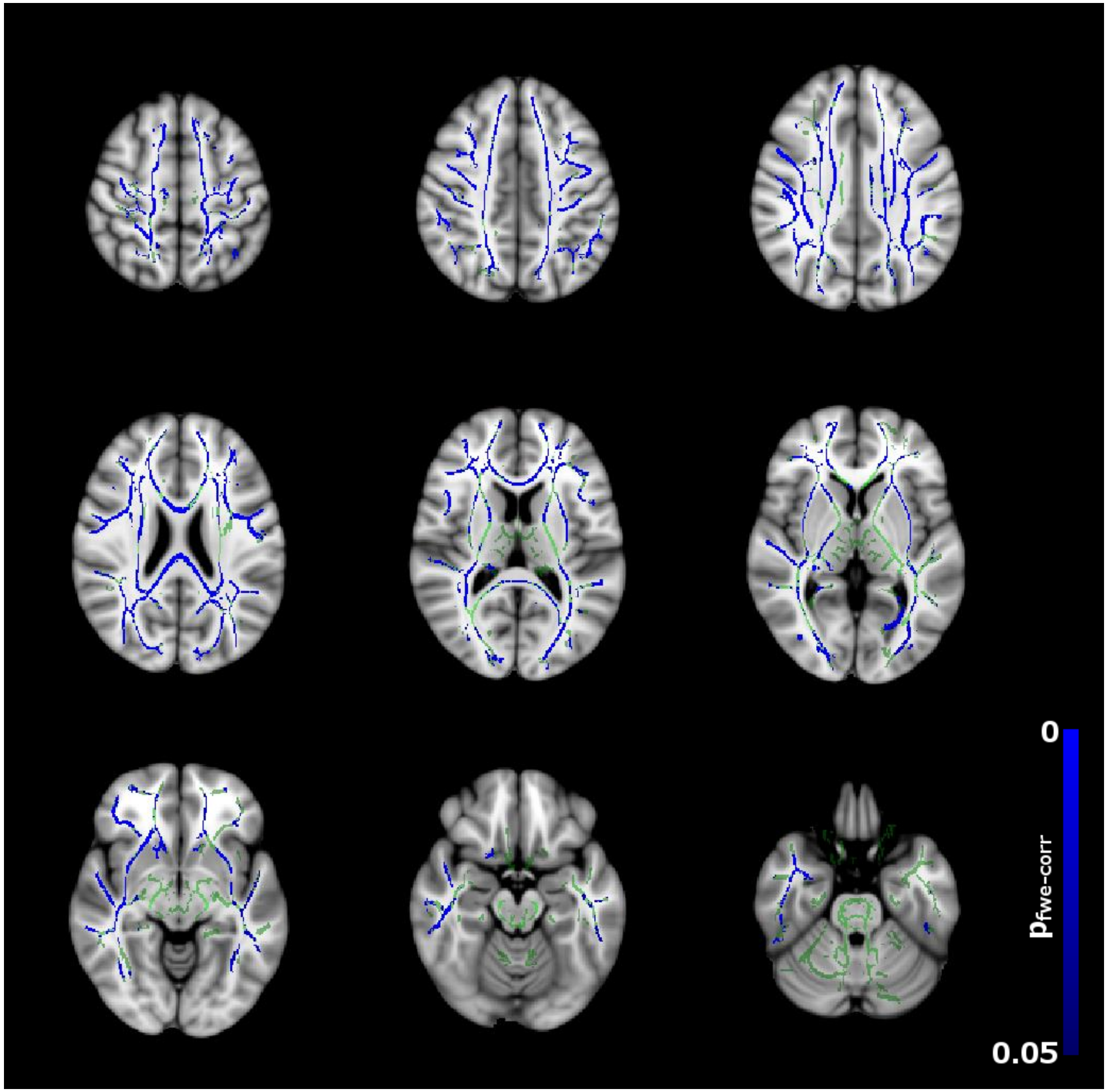

Supplementary Figure 11: Correlation between MD and age for the male subjects (p<0.05, corrected) in the 8-13 age range, corrected for FSIQ and total brain volume. Blue regions indicate a significant decrease in MD. Corresponds to figure 8 in the main text (8-16 age range).

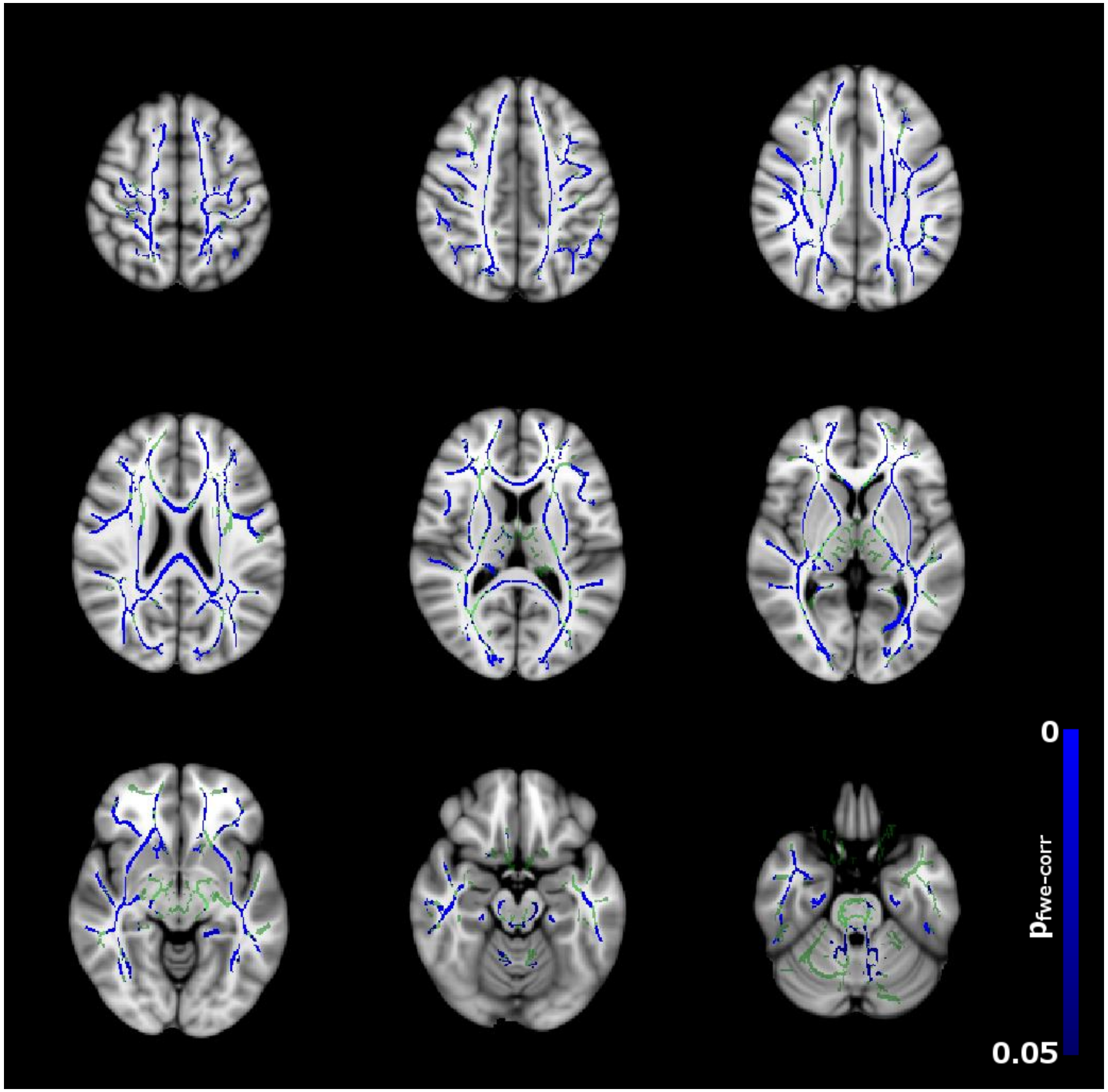

Supplementary Figure 12: Correlation between λ_{radial} and age for the male subjects ($p<0.05$, corrected) in the 8-13 age range, corrected for FSIQ and total brain volume. Blue regions indicate a significant decrease in λ_{radial}. Corresponds to figure 9 in the main text (8-16 age range).
