# Supplementary material for: Sexual Dimorphism in White Matter Developmental Trajectories Using Tract-Based Spatial Statistics
Source: Brain Connect. 2016 Feb 1;6(1):37–47. doi: 10.1089/brain.2015.0340 (PMC4744889; doi:10.1089/brain.2015.0340)
Supplement: Supplemental data [file Supp_Figure6.pdf]

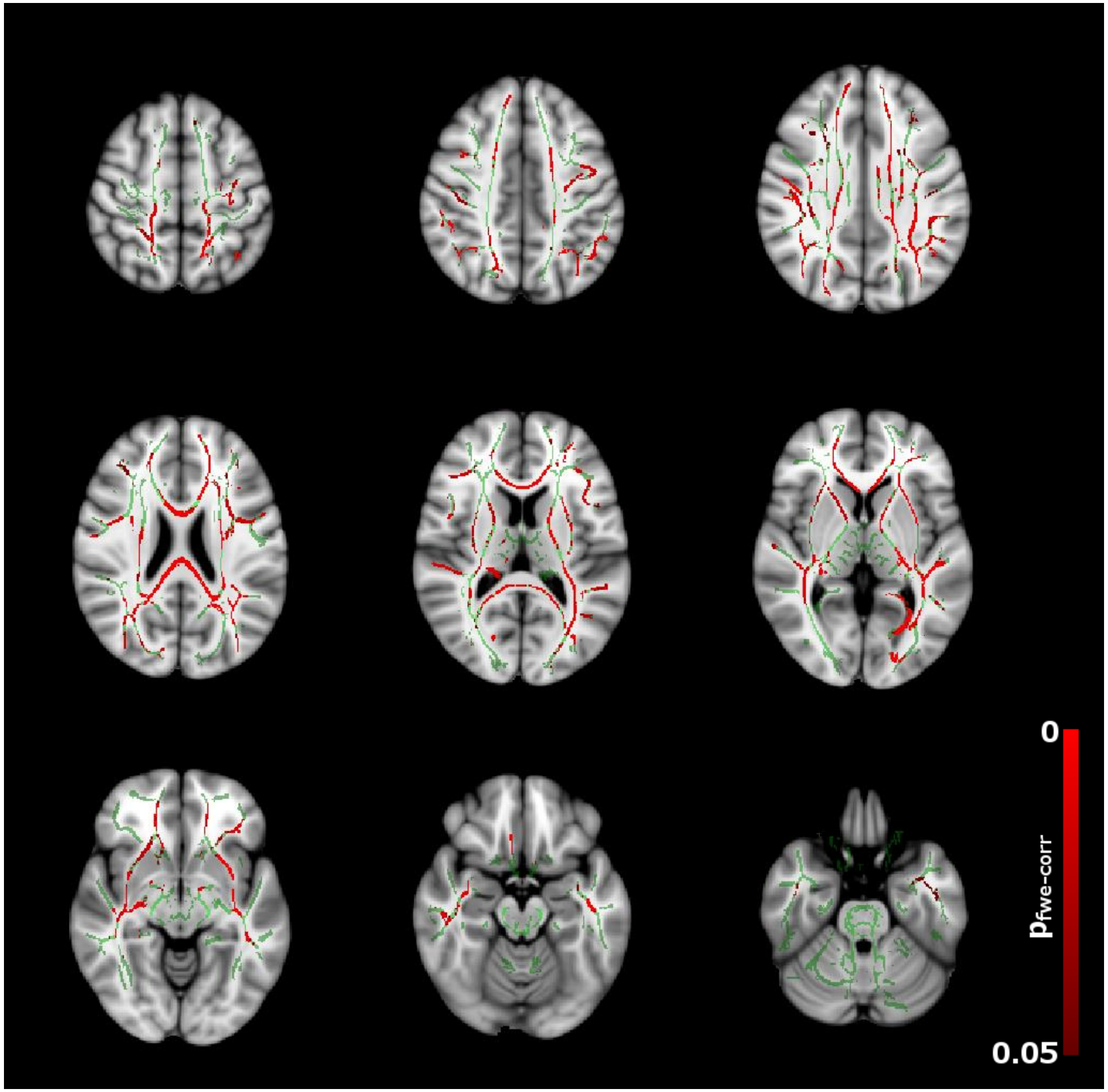

Supplementary Figure 6: Age-gender interactions in FA between male and female subjects ($p<0.05$, corrected) in the 8-13 age range, corrected for total brain volume and FSIQ. Red regions indicate a significantly steeper slope in males than females. No significant difference was seen in the 8-16 age range, although several regions were close to significance ($p<0.1$)
